# No serological association between *Porphyromonas gingivalis or Prevotella intermedia* and early spondyloarthritis: analysis from the DESIR cohort

**DOI:** 10.1007/s10238-026-02204-5

**Published:** 2026-06-22

**Authors:** Franck Zekre, Tiphany Neel, Mireille Paul, Myriam Normand, Anne Tournadre, Hubert Marotte

**Affiliations:** 1 Université Jean Monnet Saint-Etienne, CHU Saint-Etienne, Mines Saint- Etienne, INSERM, SAINBIOSE U1059 and CIRI, GIMAP Team, U111, CNRS, ENS, UCBL1, Saint-Etienne, F-42023 France; 2 CHU Saint-Etienne, Saint-Etienne, F-42023 France; 3 Université Jean Monnet Saint-Etienne, Mines Saint-Etienne, Inserm, Sainbiose, U1059 Saint-Etienne, F-42023, France; 4 Université Jean Monnet Saint-Etienne, Institut d’Administration des Entreprises de Saint-Etienne, Saint-Etienne, France; 5 Department of Rheumatology, CHU Clermont-Ferrand, UNH, INRA UMR 1019, Université Clermont Auvergne, Clermont-Ferrand, France; 6 Université Jean Monnet Saint-Etienne, CHU Saint-Etienne, Mines Saint- Etienne, INSERM, SAINBIOSE U1059, Saint-Etienne, F-42023 France

**Keywords:** Axial spondyloarthritis, *Porphyromonas gingivalis*, *Prevotella intermedia*, Periodontal disease, Oral dysbiosis, DESIR cohort

## Abstract

*Porphyromonas gingivalis* and *Prevotella intermedia* are key oral pathogens implicated in periodontal disease and have been associated with rheumatoid arthritis and specific categories of juvenile idiopathic arthritis. Whether oral dysbiosis involving *Porphyromonas gingivalis* or *Prevotella intermedia* contributes to axial spondyloarthritis pathogenesis remains unclear. We evaluated *Porphyromonas gingivalis*- and *Prevotella intermedia*-specific IgG titres across early axial spondyloarthritis phenotypes within the DESIR cohort and assessed the potential influence of smoking status. Serum samples from 554 participants in the DESIR cohort were analyzed. Anti-*Porphyromonas gingivalis* and anti-*Prevotella intermedia* IgG titres were measured using a validated in-house enzyme-linked immunosorbent assay. Patients were classified into axial spondyloarthritis, axial spondyloarthritis with psoriasis, axial spondyloarthritis with inflammatory bowel disease, undifferentiated axial spondyloarthritis, or chronic low back pain controls. Antibody titres were compared using non-parametric tests. In addition, multivariable logistic and linear regression analyses were performed adjusting for age, sex, BMI and smoking status. Anti-*Porphyromonas gingivalis* and anti-*Prevotella intermedia* IgG titres did not differ significantly across axial spondyloarthritis phenotypes or between axial spondyloarthritis subgroups and chronic low back pain controls (*Porphyromonas gingivalis*: *p* = 0.622; *Prevotella intermedia*: *p* = 0.491). These findings remained unchanged after multivariable adjustment for age, sex, BMI, and smoking status (all *p* > 0.1). Smoking status did not influence serological patterns in any group. Distributional analyses confirmed strong overlap in antibody titres across all phenotypes, with no subgroup showing a distinct P. gingivalis or P. intermedia serological signature. In this large early axial spondyloarthritis cohort, antibody responses to *Porphyromonas gingivalis* and *Prevotella intermedia* did not differ across phenotypes and showed no detectable serological association with axSpA. These findings did not support a major role of *Porphyromonas gingivalis* - or *Prevotella intermedia*–related systemic humoral responses in early axial spondyloarthritis, although they did not exclude a broader role of mucosal dysbiosis in disease pathogenesis.

## Background

Chronic periodontal disease, driven by microbial dysbiosis, contributes to inflammation and tissue destruction, with *Porphyromonas gingivalis* and *Prevotella intermedia* playing key roles. These pathogens are linked to rheumatoid arthritis and juvenile idiopathic arthritis, suggesting a systemic inflammatory impact. Axial spondyloarthritis, a group of inflammatory rheumatic diseases, shares some immunological features with rheumatoid arthritis, raising the possibility of a “periodonto–spondyloarthritis axis.” However, the relationship between periodontal disease and axial spondyloarthritis remains unclear. This study explores the role of *Porphyromonas gingivalis* and *Prevotella intermedia*-specific IgG titres in early axial spondyloarthritis using data from the DESIR cohort, aiming to clarify their involvement in axial spondyloarthritis pathogenesis.

## Introduction

Chronic periodontal disease is a common inflammatory condition of the oral cavity that leads to progressive destruction of the tooth-supporting apparatus. Clinically, it is characterized by gingival inflammation, bleeding, increased tooth mobility, alveolar bone loss, and ultimately tooth loss [1]. The prevalence of periodontal disease increases with age, but local risk factors, such as microbial dysbiosis, play a critical role. In particular, *Porphyromonas gingivalis* (*Pg*) and *Prevotella intermedia* (*Pi*) are over-represented in the gingival biofilm of patients with periodontitis [2]. These organisms activate innate and adaptive immune responses and promote the secretion of pro-inflammatory cytokines including interferon-γ, tumour necrosis factor (TNF), interleukin (IL)-1, IL-6, and IL-17. This cytokine cascade drives the production of metalloproteinases (MMPs), leading to extracellular matrix degradation of the gingival and periodontal tissues, and alveolar bone resorption [3].

Beyond local tissue damage, periodontal dysbiosis has been implicated in systemic inflammatory diseases. The association between periodontal disease and rheumatoid arthritis (RA) is now well documented, particularly through the induction of anti-citrullinated peptide antibodies [4]. Several interventional studies have suggested that improving periodontal status or targeting *Pg* may reduce RA disease activity [5, 6]. In addition, high IgG titres against *Pg* have been reported in specific categories of juvenile idiopathic arthritis subsets [7].

Spondyloarthritis (SpA) encompasses a group of chronic inflammatory rheumatic diseases including axial (ax)SpA, psoriatic arthritis, arthritis associated with inflammatory bowel disease (IBD), reactive arthritis, and undifferentiated axSpA. These entities share clinical, radiographic, and immunological features, including the involvement of key cytokines such as TNF, IL-1, IL-6, and IL-17 [8]. Given these overlaps with RA and the strong immunological interplay between mucosal surfaces and systemic inflammation, a potential “periodonto–spondyloarthritis axis” has been proposed. However, available data are inconsistent. Some studies have reported a higher prevalence of periodontitis in patients with ankylosing spondylitis or non-radiographic axSpA, whereas others found no clear association between periodontal disease and axSpA [9, 10].

The French DESIR cohort prospectively enrolled patients with recent-onset chronic back pain suggestive of axSpA and followed them longitudinally, allowing robust phenotypic classification after several years of follow-up [11]. This cohort offers a unique opportunity to explore early immunological signals potentially linking oral dysbiosis to different axSpA phenotypes. In the present study, we used *Pg* and *Pi* specific IgG serologies as indirect biomarkers of chronic periodontal exposure and inflammation in patients from the DESIR cohort.

Our objective was to compare anti-*Pg* and anti-*Pi* IgG titres across early axSpA subtypes and a chronic low back pain (CLBP) control group, and to assess the potential influence of smoking status as a confounder. We hypothesized that, if *Pg*- or *Pi*-driven oral dysbiosis contributed to axSpA pathogenesis, higher antibody titres would be observed in specific axSpA phenotypes compared with CLBP controls.

## Methods

### Study population

This study used serum samples from the second time point of the French DESIR cohort (ClinicalTrials.gov NCT01648907), a prospective longitudinal study of patients with recent-onset chronic back pain suggestive of axSpA. The DESIR cohort enrolled adults aged 18–50 years with inflammatory back pain lasting more than three months and less than three years. Final diagnostic confirmation was established after three years of follow-up according to ASAS criteria.

Among the 554 available serum samples, patients fulfilling the ASAS classification criteria for axSpA were included. Additional phenotypic subgroups were defined based on established disease associations recorded at baseline: axSpA, axSpA with psoriasis, axSpA with IBD, and undifferentiated axSpA. A control group of patients with CLBP and no evidence of axSpA served as controls. This study was in line with the current Good Clinical Practice Guidelines (French version) and received approval from the appropriate ethics committee. All patients gave their written informed consent. A website containing the detailed description of the centres, the organization of the cohort and the full detailed protocol and Case Record Form is at http://www.lacohortedesir.fr. The biological resources centre (Paris, Bichat Hospital Claude Bernard – CRB BCB, Certificate number 34457, S. Tubiana) was in charge of centralizing and managing biological data collection.

## Serological assays

Anti-*Pg* and anti-*Pi* IgG titres were measured using an in-house enzyme-linked immunosorbent assay (ELISA), as previously described [7]. *Pg* strain ATCC 33,277 and *Pi* CIP 103,607 were cultured anaerobically on non-selective sheep blood agar supplemented with 0.0002% menadione sodium bisulfite and 0.4% hemin chloride for 7 days at 37 °C. Bacterial suspensions were then heat-inactivated (60 °C for 45 min), filtered (0.22 μm), diluted (1:10), and used to coat 96-well ELISA plates overnight at 4 °C.

Human plasma was diluted 1:900 in PBS containing 1% BSA and incubated in duplicate for 2 h at room temperature. After washing, plates were incubated with peroxidase-conjugated goat anti-human IgG H + L (Jackson ImmunoResearch, West Grove, PA) (diluted 1:50,000 in PBS) for 2 h at room temperature. Following a final wash, detection was achieved by tetramethylbenzidine substrate (R&D Systems, Minneapolis, MN). The reaction was stopped by the addition of a 1M H_2_SO_4_ solution, and absorbances were measured at 450 nm.

Seropositivity cut-offs were established as 95th percentile of optical density values obtained from 27 healthy blood donors. For each plate, a six-point calibration curve was generated using serial dilutions (1:100 to 1:16,200) of a pool of positive serum to correct for inter-plate variability. High and low positive controls were included in every run. Titres were expressed in arbitrary units (AU), defined by reference to the calibration curve.

### Statistical analysis

Statistical analyses were performed using R software. Antibody titres were first compared between the overall axSpA group and CLBP controls, and then across axSpA phenotypic subgroups using non-parametric Kruskal-Wallis tests.

To account for potential confounders, multivariable linear regression analyses were performed after Box-Cox transformation of antibody levels, including age, sex, BMI, smoking status, and disease group (SpA vs. CLBP) as covariates. Logistic regression models were also conducted to assess the association between antibody levels and disease status, adjusted for the same variables.

Data visualization included box plots representing median and interquartile ranges, as well as kernel density curves modelling antibody titre distribution across groups. Statistical significance was set at *p* < 0.05.

## Results

A total of 554 serum samples from the DESIR cohort were analysed. Patients were classified into five groups: axSpA (*n* = 126), axSpA with PsA (*n* = 101), axSpA associated with IBD (*n* = 36), undifferentiated axSpA (*n* = 241), and a CLBP control group (*n* = 50). Demographic and clinical characteristics are summarised in Table 1. The median age of the axSpA group was 30 years [18.5–50.4], while the median age for the CLBP control group was 35.7 years [18.2–50.1]. In the axSpA group, 58.7% of patients were male, compared to 36% in the CLBP group. In the axSpA with PsA group, 44.6% were male, while 38.9% of the axSpA with IBD group and 42.3% of the undifferentiated axSpA group were male. Body mass index (BMI) varied across groups, with a median BMI of 22.8 [16.7–34.6] in the axSpA group and 22.4 [17.3–32.8] in the CLBP group. The axSpA with PsA group had a notably higher median BMI of 24.4 [16.3–48.9]. Regarding smoking status, 35.7% of axSpA patients were smokers, 39.6% in the axSpA with PsA group, 38.9% in the axSpA with IBD group, 33.6% in the undifferentiated axSpA group, and 26% in the CLBP group. The duration of symptoms was comparable across groups, with a mean duration of approximately 1.5 years for all groups.

**Table 1:** Demographic and clinical characteristics of patients analysed from the DESIR cohort

	axSpA	Undifferentiated SpA	SpA with PsA	SpA with IBD	CLBP
	(*n* = 126)	(*n* = 241)	(*n* = 101)	(*n* = 36)	(*n* = 50)
**Age**, median [min-max]	30 [18.5–50.4]	34.6 [18.1–60.6]	33.9 [18-49.6]	32.9 [18.8–49]	35.7 [18.2–50.1]
**Male**, n (%)	74 (58.7)	102 (42.3)	45 (44.6)	14 (38.9)	18 (36)
**BMI**, median [min-max]	22.8 [16.7–34.6]	23.2 [16.5–38]	24.4 [16.3–48.9]	24 [18.3–34.8]	22.4 [17.3–32.8]
**Smoking**, n (%)	45 (35.7)	81 (33.6)	40 (39.6)	14 (38.9)	13 (26)
**Duration of symptoms in years**,					
**mean ± SD**	1.65 ± 0.88	1.5 ± 0.8	1.5 ± 0.85	1.5 ± 1.23	1.5 ± 0.9

axSpA: axial spondyloarthritis; PsA: psoriatic arthritis; IBD: inflammatory bowel disease;  CLBP: Chronic low back pain; SD: standard deviation

## Anti–P. gingivalis and anti–P. intermedia IgG titres across SpA phenotypes

**Fig. 1:**
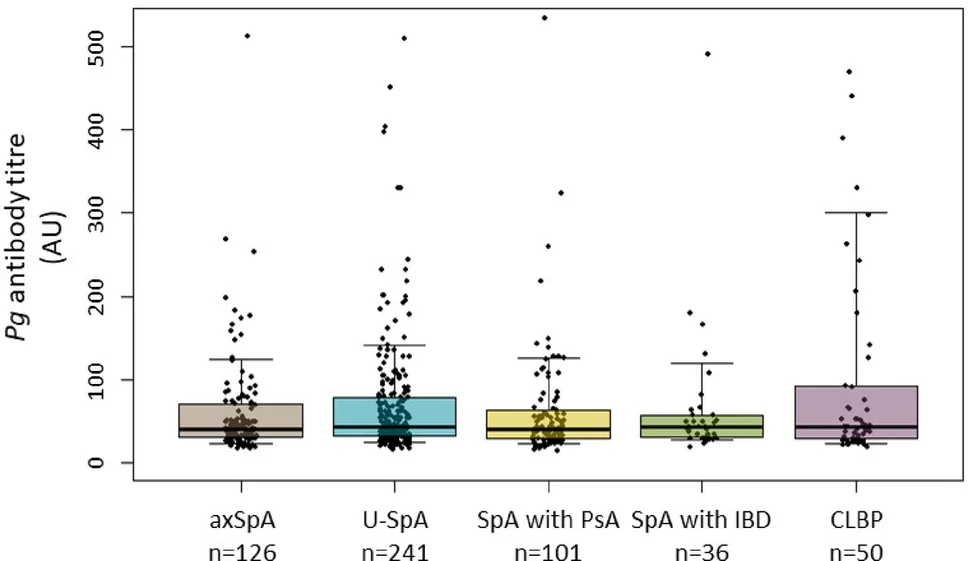
Anti-Porphyromonas gingivalis IgG titres across axial spondyloarthritis phenotypes and chronic low back pain controls The figure shows the distribution of Porphyromonas gingivalis antibody titres for the following groups: axial spondyloarthritis (n=126), axial spondyloarthritis with psoriatic arthritis (n=101), axial spondyloarthritis associated with inflammatory bowel disease (n=36), undifferentiated axial spondyloarthritis (n=241), and chronic low back pain (n=50). No significant differences were observed in Porphyromonas gingivalis antibody titres between groups (Kruskal-Wallis p=0.622). The data are presented as single value with box plots showing the median and with interquartile ranges. Pg: Porphyromonas gingivalis; AU: arbitrary units; axSpA: axial spondyloarthritis; PsA: psoriatic arthritis; IBD: inflammatory bowel disease; U: undifferentiated; CLBP: chronic low back pain

**Fig. 2:**
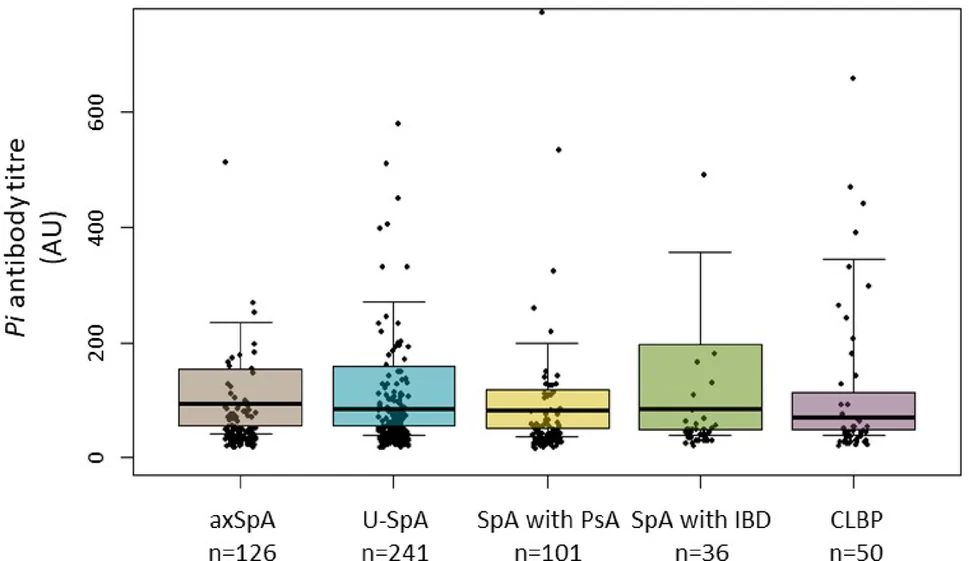
Anti-Porphyromonas gingivalis IgG titres across axial spondyloarthritis phenotypes and chronic low back pain controls The figure shows the distribution of Porphyromonas gingivalis antibody titres for the following groups: axial spondyloarthritis (n=126), axial spondyloarthritis with psoriatic arthritis (n=101), axial spondyloarthritis associated with inflammatory bowel disease (n=36), undifferentiated axial spondyloarthritis (n=241), and chronic low back pain (n=50). No significant differences were observed in Porphyromonas gingivalis antibody titres between groups (Kruskal-Wallis p=0.622). The data are presented as single value with box plots showing the median and with interquartile ranges. Pg: Porphyromonas gingivalis; AU: arbitrary units; axSpA: axial spondyloarthritis; PsA: psoriatic arthritis; IBD: inflammatory bowel disease; U: undifferentiated; CLBP: chronic low back pain

As shown in Fig.1 , there were no significant differences in anti-*Pg* IgG titres between SpA and CLBP controls, nor across the five axSpA groups (Kruskal-Wallis *p* = 0.622). Similarly, as shown in Fig.2 , anti-*Pi* IgG titres did not differ between axSpA and CLBP control, or between axSpA subgroups and CLBP controls (Kruskal-Wallis *p* = 0.491). Median values and distribution shapes were comparable across all phenotypes, and no SpA subtype exhibited a distinct serological profile.

### Multivariable analyses

In multivariable linear regression analyses adjusted for age, sex, BMI, and smoking status, no significant association was observed between disease group (SpA vs. CLBP) and antibody levels (anti-Pg: *p* = 0.51; anti-Pi: *p* = 0.41). Similar results were obtained in logistic regression models, with no significant association between antibody levels and disease status.

## Discussion

In this large early axSpA cohort, we found no significant difference in IgG titres against *Pg* or *Pi* across the main axSpA phenotypes or when compared with a chronic low back pain control group. These findings suggest that, unlike in RA, antibody responses to *Pg* and *Pi* did not distinguish early axSpA phenotypes and did not support a major contribution of Pg/*Pi*-related systemic humoral responses in early axSpA, including non-radiographic axSpA.

The absence of a *Pg*/*Pi* signature in early axSpA contrasts sharply with RA, where *Pg* is strongly implicated in disease initiation through its capacity to drive protein citrullination and break immune tolerance [2, 4–6]. Elevated anti-*Pg* titres have also been reported in specific juvenile idiopathic arthritis categories, such as enthesitis-related arthritis [7], reinforcing the relevance of oral dysbiosis in some forms of inflammatory arthritis.

In SpA, however, prior studies have yielded inconsistent findings. Some reports described a higher prevalence of periodontitis in ankylosing spondylitis (AS) or SpA cohorts, especially in populations with significant spinal stiffness, raising concerns for confounding by poor oral hygiene [12, 13]. Conversely, several more recent case-control studies failed to confirm such associations [14, 15], which aligns with our present serological results and suggests that these inconsistences may be due to differences in study design or population characteristics.

In addition to these prior studies, large-scale epidemiological datasets have provided mixed signals. In the UK Biobank, self-reported AS correlated with oral ulcers but not with objective markers of periodontitis [16]. In a Korean cohort, *Pg* prevalence and periodontal severity did not differ between AS patients and controls, although worse spinal mobility was associated with poorer periodontal status, and both periodontal treatment and anti-TNF therapy improved oral parameters [17]. These observations underscore the difficulty in disentangling true immunopathological links from functional or behavioural confounders (reduced mobility, limited oral hygiene).

Taken together, our data did not support a major contribution of *Pg*- or *Pi*-driven systemic humoral responses to the early immune disturbances underlying SpA. This result is notable given the strong immunological convergence between periodontal inflammation and SpA-particularly IL-17-driven mucosal responses, osteoimmunology, and neutrophil-mediated tissue damage. While *Pg* and *Pi* are key triggers of periodontal inflammation, their serological imprint does not appear to reflect or influence the early pathogenic processes of axSpA in this well-defined cohort.

It is possible that other components of the oral or gut microbiome, rather than *Pg*/*Pi* specifically, could contribute to mucosal immune activation in SpA. Emerging metagenomic studies have identified altered populations of bacteria in the oral and intestinal microbiomes of SpA patients, suggesting complex interactions among multiple microbial species rather than a singular pathogen effect. Our *Pg*/*Pi*-focused serological approach may therefore underestimate the breadth of microbial exposures relevant to SpA pathogenesis.

Strengths of this study include the large sample size, robust methodology, and the use of a well-characterized early axSpA cohort, standardized phenotyping after 3 years of follow-up, and calibrated ELISA assays. Importantly, our results remained unchanged after adjustment for major confounders, including age, sex, BMI and smoking status, supporting the robustness of the observed absence of association. These findings suggest that the absence of difference in antibody titres between groups is not explained by differences in demographic or clinical characteristics.

Although exposure to biologic therapies was likely limited at this early stage of the DESIR cohort, we cannot exclude that treatment-related modulation of immune responses may have attenuated potential differences between groups.

Several limitations must be acknowledged. First, periodontal status was not clinically assessed. *Pg*/*Pi* serologies provide indirect measures of systemic immune responses to chronic periodontal exposure and do not capture local periodontal inflammation or disease severity. Second, antibody titres were measured at a single time point, precluding assessment of longitudinal relationships between periodontal exposure and disease evolution. Third, treatment exposure at the time of sampling was not available in sufficient detail to allow robust adjustment. Although patients were included at an early stage of the DESIR cohort, some axSpA patients were likely receiving NSAIDs or biologic therapies, including TNF inhibitors, which may influence both periodontal inflammation and systemic antibody responses, potentially attenuating differences between groups. In addition, the relatively young age of the cohort (median approximately 30 years) and the early disease stage may have limited the ability to detect associations with periodontal disease, which is generally cumulative and more prevalent in older populations. The sample sizes of certain subgroups, particularly patients with axSpA associated with inflammatory bowel disease, were also relatively small, potentially reducing statistical power for subgroup analyses. Another limitation relates to the targeted nature of our approach. Dysbiosis is increasingly recognized as a community-level phenomenon rather than being driven by individual pathogens. Restricting the analysis to *Pg* and *Pi* may therefore underestimate the contribution of broader oral or gut microbiome alterations to axSpA pathogenesis.

Finally, the CLBP group does not represent a healthy control population, as all participants were initially recruited within the DESIR cohort for recent inflammatory back pain suggestive of SpA. While this may have reduced the contrast between groups, it also constitutes a methodological strength, as it enabled comparison between patients with similar initial clinical presentations, thereby limiting selection bias and reflecting real-world diagnostic uncertainty in early disease. In this context, the absence of differences in antibody titres further supports the lack of specificity of Pg- and Pi-directed humoral responses in axSpA.

## Conclusion

In conclusion, this study found no detectable serological association between *Pg* or *Pi* and early axial spondyloarthritis, arguing against a major contribution of *Pg*/*Pi*-related systemic humoral responses in early axSpA. However, these findings do not exclude a role for broader, community-level mucosal dysbiosis. Future longitudinal studies integrating clinical periodontal assessment and comprehensive microbiome profiling will be essential to clarify the role of mucosal environments in axSpA pathogenesis.

## Data Availability

The datasets analysed during the current study are available from the corresponding author on reasonable request.

## Abbreviations

Pg: *Porphyromonas gingivalis *
Pi: Prevotella Intermedia
TNF: Tumour necrosis factor
IL: Iinterleukin
MMPs: Metalloproteinases
RA: Rheumatoid arthritis
SpA: Spondyloarthritis
axSpA: Axial SpA
IBD: Inflammatory bowel disease
CLBP: Chronic low back pain
ELISA: Enzyme-linked immunosorbent assay
AU: Arbitrary units
BMI: Body mass index
